# Analysis of skin conductance response during evaluation of preferences for cosmetic products

**DOI:** 10.3389/fpsyg.2015.00103

**Published:** 2015-02-09

**Authors:** Hideki Ohira, Naoyasu Hirao

**Affiliations:** ^1^Department of Psychology, Nagoya UniversityNagoya, Japan; ^2^Research Center, Shiseido Co. Ltd.Yokohama, Japan

**Keywords:** preference, marketing, skin conductance response, paired comparison, cosmetic products

## Abstract

We analyzed skin conductance response (SCR) as a psychophysiological index to evaluate affective aspects of consumer preferences for cosmetic products. To examine the test-retest reliability of association between preferences and SCR, we asked 33 female volunteers to complete two experimental sessions approximately 1 year apart. The participants indicated their preferences in a typical paired comparison task by choosing the better option from a combination of two products among four products. We measured anticipatory SCR prior to expressions of the preferences. We found that the mean amplitude of the SCR elicited by the preferred products was significantly larger than that elicited by the non-preferred products. The participants' preferences and corresponding SCR patterns were well preserved at the second session 1 year later. Our results supported cumulating findings that SCR is a useful index of consumer preferences that has future potential, both in laboratory and marketing settings.

## Introduction

Evaluating consumer preferences is a critical element of marketing. Traditionally, preference is measured using psychological indexes, such as ratings, semantic differential scales, and conjoint analysis (Netzer et al., 2008). However, it is generally difficult to use psychological and behavioral indexes to measure preferences for products for which there is a high degree of consumer interest but a relatively small degree of difference among the products in terms of performance and quality. This is because preference for a product is not solely determined by cognitive processes but also by affective and intuitional responses to the products (Bagozzi et al., 1999). Cosmetics are such products. While cosmetics are often considered to be necessities for women, especially basic cosmetics such as emulsions, functional qualities of cosmetics have been thoroughly asserted in cosmetic advertising (Kumar, 2005). As consumers tend to make affective and intuitional choices regarding such products, ways of measuring preference not only in cognitive aspects but also affective aspects have value when developing marketing strategies.

Based on this motivation, several researchers have developed methods for measuring preferences for products and brands using physiological and neural responses (e.g., Venkatraman et al., 2012; Javor et al., 2013). Wang and Minor (2008) summarized the validity and reliability of multiple physiological indexes, including EEG, hemispheric lateralization, pupillary response, electro-dermal response, voice pitch, heart rate, vascular activity, facial muscle activity, eye movement, and brain imaging, using available previous findings. They concluded that electro-dermal activity, facial muscle activity, and brain imaging constitute effective indexes for evaluating affective facets of preference, such as pleasure and arousal (also see Figner and Murphy, 2011). Among those indexes, electro-dermal activity is the easiest to measure and perhaps the most robust, making it very useful both in laboratory and marketing settings. For example, both skin conductance response (SCR; an index of electro-dermal activity) and startle reflex of eyeblink are consistently larger for disliked brand names compared with liked brand names (Walla et al., 2011).

Most previous studies have adopted a relatively simple experimental paradigm where stimuli are intermittently presented to participants to measure physiological responses corresponding to rating of preference or buying intentions. Usually, paired comparison can provide more reliable and rigorous psychometric data for preferences, with lesser effort for participants, comparing rating and rank ordering of preferences. Thus, combination of psychophysiological responses reflecting affective facets of preferences and paired comparison indicating cognitive facets of preferences can be a promising tool for marketing. Recently, Ahn and Picard (2014) proposed such an experimental paradigm where affective facets of preferences are measured by facial expressions of participants and cognitive facets of preferences are measured by self-reports of participants, and showed that this paradigm can predict success of marketing of real products. Comparing to facial expressions, merits of SCR as an index of preference should be that standard methodologies of measurement and analysis of SCR have been established, and that physiological background and neural basis of SCR have been clarified (Figner and Murphy, 2011). Therefore, to examine utility of SCR as a psychophysiological index of affective facets of preferences, we analyzed patterns of SCR during evaluation of preferences for cosmetic products using Thurstone's paired comparison, wherein we presented all possible pairs of two cosmetic products for participants to judge which stimulus they preferred. To measure SCR during the task such that after the presentation of a pair of cosmetic products, one of the products was presented again, and the participants were asked whether they desired the product more or less than the other product from the pair. Using this paradigm, we measured anticipatory SCR, which precedes and presumably, predicts following explicit behavioral expressions of preference for a specific product. As SCR usually shows relatively rapid habituation, examination of the test-retest reliability of the association between cognitive preferences and SCR should be helpful to evaluate preferences of products in relatively long time scales. For this purpose, we required participants to complete the same experimental session twice with an interval of approximately a year. This enabled us to examine the test-retest reliability of participant preferences and the corresponding sympathetic responses.

## Materials and methods

### Participants

Thirty-three healthy native Japanese women (age; *M* = 40.7, *SD* = 5.1) participated in this experiment. All of the participants provided written informed consent. This study was approved by the ethical committee at Nagoya University. We did not check handedness of each participant however we do not consider that this is a critical issue because any motor responses such as reaction time were not measured in this study.

### Stimuli and procedure

We used four commercially available types of emulsions, which is a product meant to protect skin from ultraviolet sunlight during daytime, as stimuli. The four products were all produced by different Japanese cosmetic companies and retailed at nearly the same price (approximately 3000 Japanese Yen: concrete information about the products and brans is not shown considering conflicts of interests). Prior to the experiment, we asked the participants whether they were familiar with the product brands. The percentage of participants who were familiar with each brand is as follows: Product A (87.9%), Product B (81.8%), Product C (100%), and Product D (57.6%). We presented visual images of the containers and outer packages of the products (see Supplemental Material) in a size of 1 m × 1 m on a screen located 2.5 m in front of the participants (visual angle, approximately 23°). The image was generated by a projector connected to a computer. The experimental sessions were conducted in a comfortable air-conditioned room (24–27°C).

**Figure 1:**
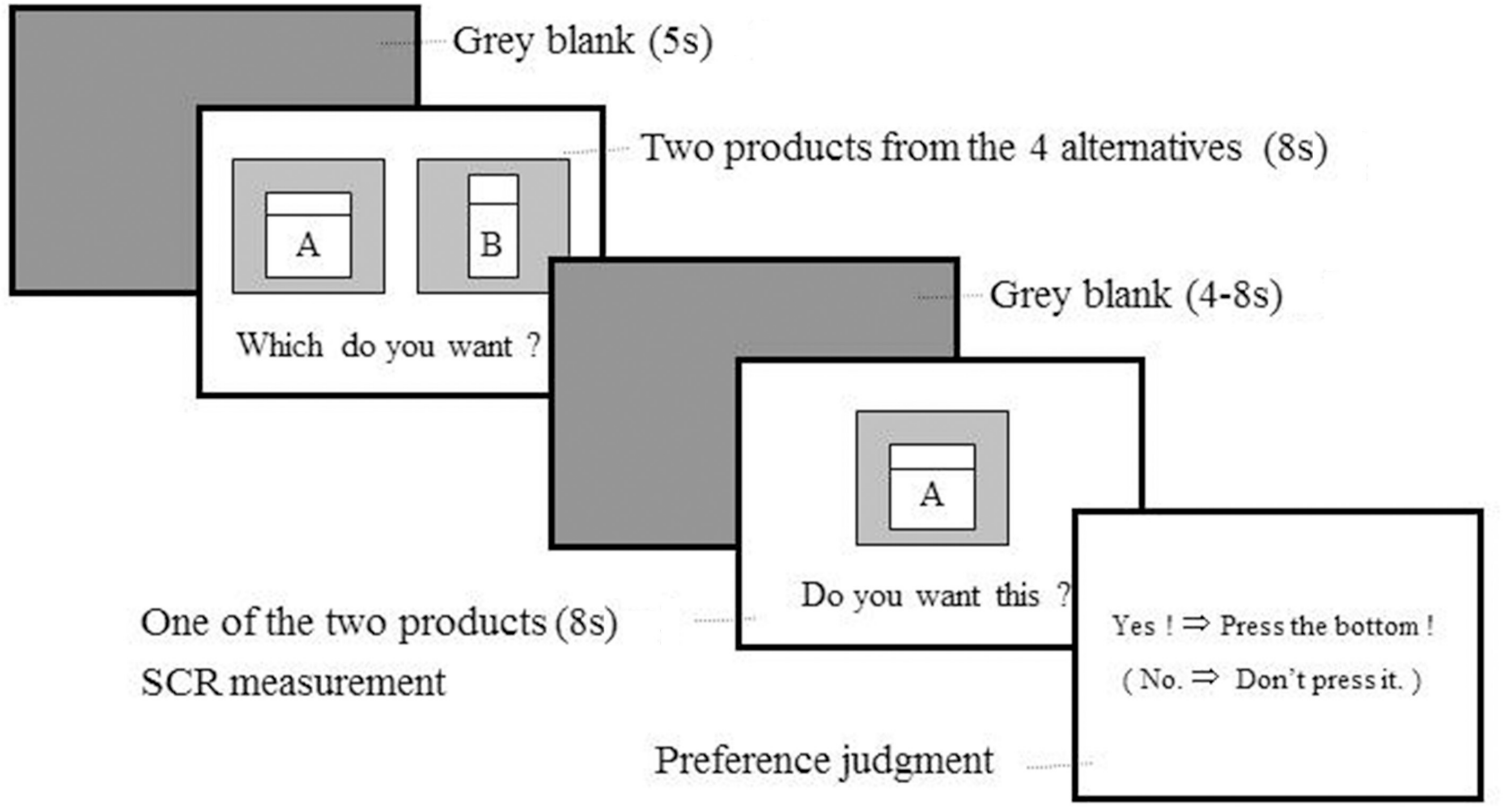
**Time sequence of a trial in the preference judgment task**. First, the participant was shown images of two cosmetic products selected from four options. Then, one of the two products was presented and the participant pressed a button with her right hand if she considered the presented product to be more preferable and desirable than the other product. Each participant completed two sequences of 13 trials in this task (26 trials in total). The trial order was randomized. The behavioral and SCR data from the 1st and 13th trials were discarded from the analyses to reduce the influence of the primary and final effects. We measured SCR continuously during the experimental session and analyzed SCR data from the interval between 0 and 8 s from stimulus onset in the judgment period.

After providing informed consent, between one and five participants simultaneously completed the experimental sessions. Participants were not acquaintances each other, and also they had no social interactions during the experimental sessions. Specifically, they did not talk each other, and did not see other participants' performance of the task, during the experimental sessions. First, the participants saw the images of the four products and were given several minutes to read a brief description that contained the bland names, product names, and basic features of each product. No information about the prices of the products was given. Then, the participants were asked to complete a task wherein they evaluated their preference for each stimulus (Figure 1). Each task trial began with a gray-blank screen for 5 s, and then two of the four stimuli were presented at the right and left sides of the screen for 8 s. The participants were asked to consider the products and decide which they would be interested in purchasing. Then, the gray-blank screen was shown for a jittered period that ranged between 4 and 8 s, followed by one of the two stimuli for 8 s. During this period, we measured SCR as an index of sympathetic nervous system activity. Afterwards, a response signal was given to cue the participant to press a button by their right hand if they desired the currently presented product more than the absent member of the stimulus pair. If they preferred the other product, they were instructed to make no response. For every combination of two products from the four products (12 combinations), each of the two products was presented once. Thus, the participants completed 24 trials in total. In other words, we evaluated preference for each product six times. All of the participants completed the experimental session twice with an interval of approximately a year, in 2011 and 2012. The experimental procedure was exactly identical in both sessions. At the end of each experimental session, the participants were thanked and given 4000 Japanese Yen as compensation.

### Skin conductance response

We measured SCR using EDA amplification equipment (DA-3, Vega Systems Co. Ltd., Tokyo, Japan). The electrodes for measurement of SCR were attached to second phalanxes of the mid and index fingers of the left hands of the participants. We used an A/D translator (PC data logger U12, LabJack Co. Ltd., Lakewood, CO, USA) to convert the analog SCR signal to a digital signal. This was done with a sampling rate of 10 Hz, a time constant of 4 s, and a low-pass filter of 5 Hz, and recorded in a personal computer (PCG-81214N, Sony Co. Ltd., Tokyo, Japan). After the experimental sessions, the SCR data were analyzed offline. We calculated the mean SCR amplitudes (μS) from 2 to 6 s during the 8 s product presentation period (prior to preference judgment). We used the level of skin conductance at the onset of product presentation as the baseline and subjected the data to statistical analysis as follows.

### Statistical analysis

We compared SCR amplitudes between trials where participants preferred the presented product (preferred condition) and trials where they preferred the absent product (non-preferred condition). We also examined the effect of repeating the task on SCR (test-retest reliability). Thus, we conducted a 2 (Preference: preferred vs. non-preferred) × 2 (Session: first vs. second) repeated-measures analysis of variance (ANOVA) for SCR amplitudes. Our acquired SCR data had a Poisson-like distribution pattern, so we added 0.23 to the original SCR amplitude values and then transferred these data to logarithmic values, as the minimum SCR value was −0.224 μS.

For a behavioral index, we calculated the rate of preference judgments. Specifically, the rate that a participant judged as she preferred a product to another product in 6 trials of paired comparison was calculated for each product. Also, on the basis of the standard method of Thurstone's paired comparison, a scale value of each product was determined by the inverse function of the normal distribution (*z*-transformation) of a mean of the rate of preference judgment. To confirm the reliability of the participant preferences between the first and second experimental sessions, we calculated the rate of preference judgments for each product. We conducted a 4 (Product: A–D) × 2 (Session: first vs. second) repeated-measures ANOVA for the rates of preference judgments. Furthermore, we tested association of preferences of products and SCR values, measured in the paradigm of the present study. For this aim, a correlation between the *z* scores of preference judgment and the log-transformed SCR values for the 4 products, per each participant. The correlation coefficients were transformed to *z* values using Fischer's method, and then were subjected to one-sample *t*-tests, in the first and second experimental sessions, separately. A significant result of this *t*-test means that preferences and SCR statistically significantly correlate each other.

## Results

**Figure 2:**
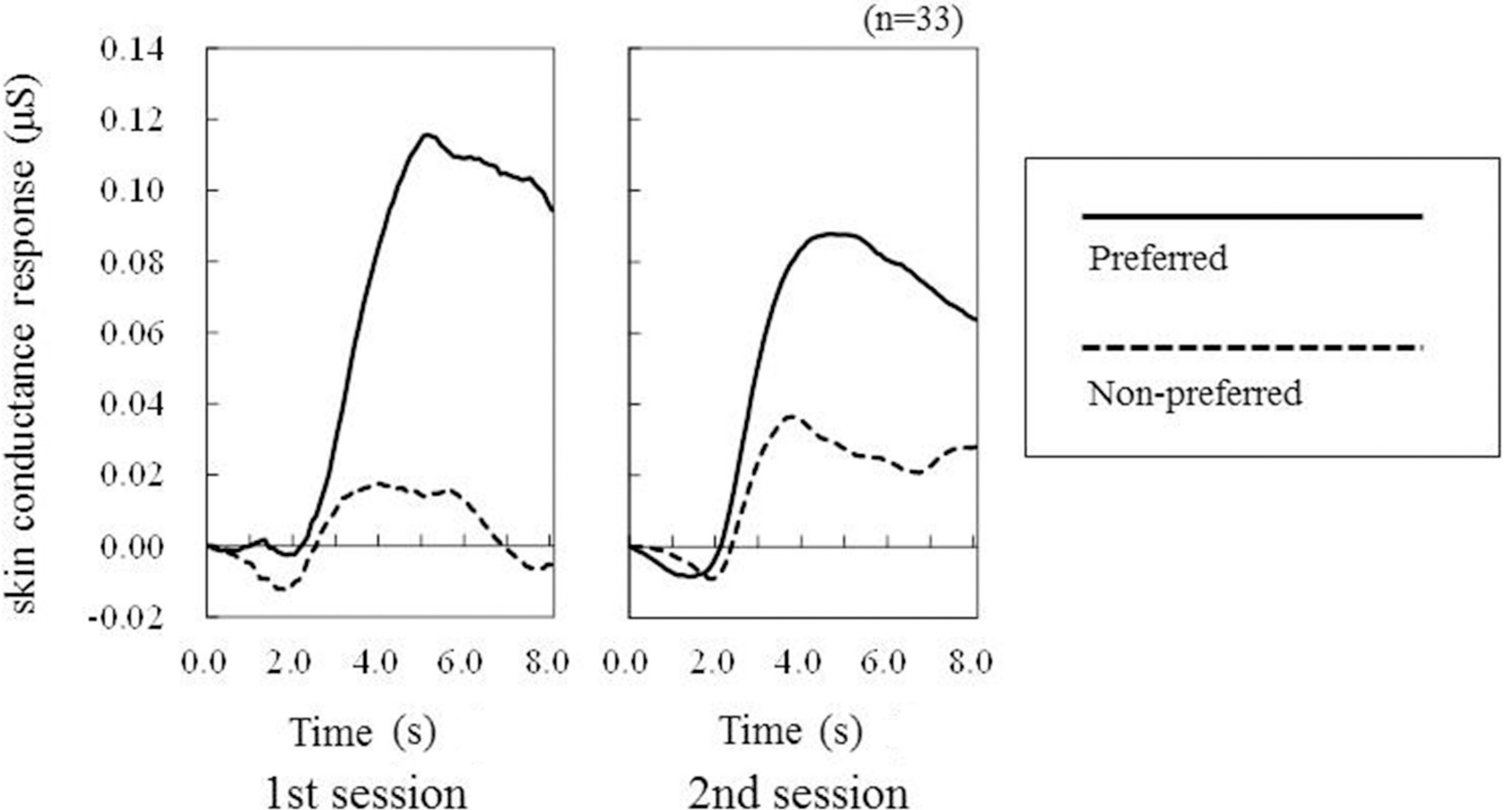
**Grand averaged SCR waveforms elicited by preferred products and non-preferred products**.

**Figure 3:**
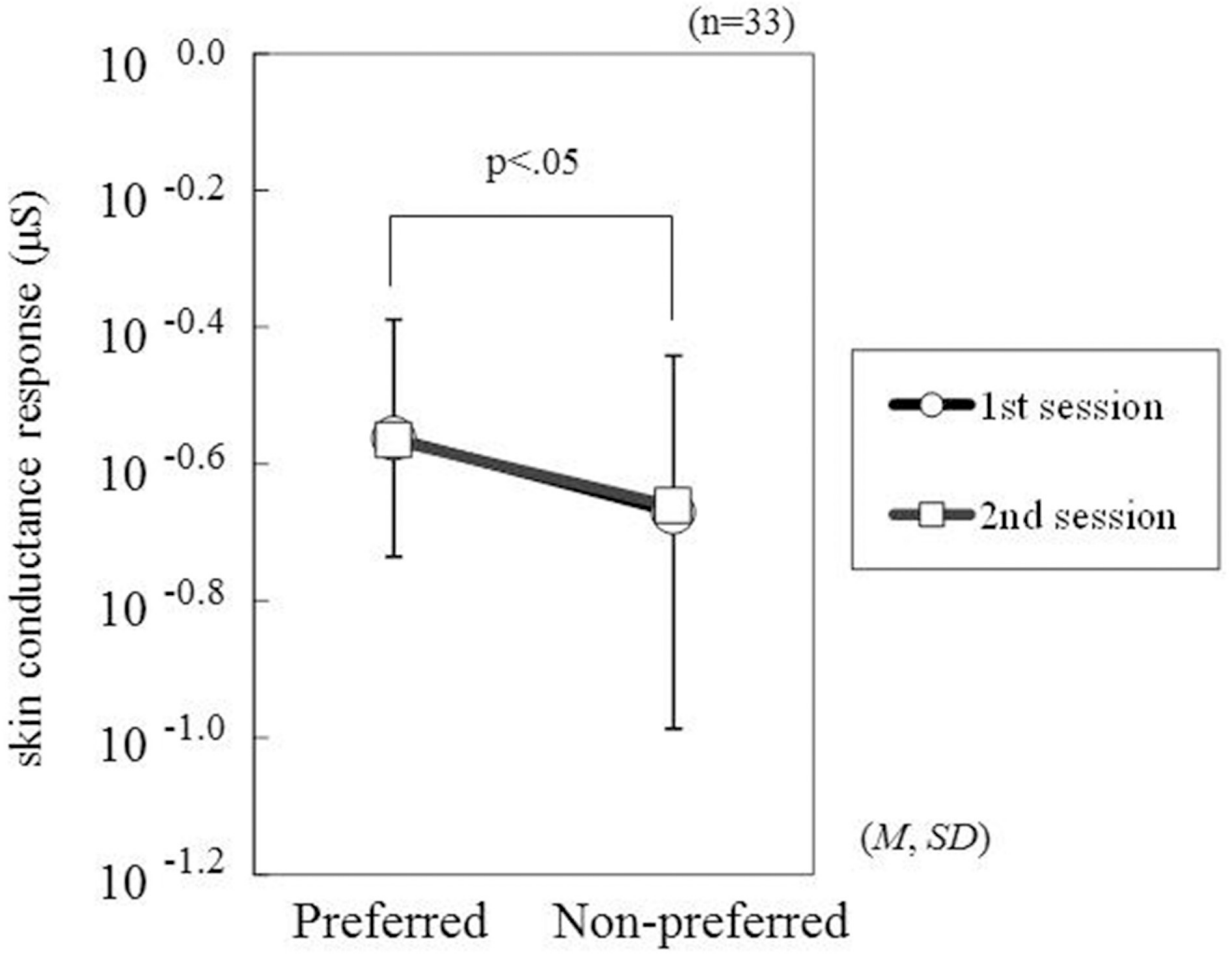
**Means and standard deviations of SCR amplitudes elicited by preferred products and non-preferred products**. The values of the SCR amplitudes were converted to logarithmic scores after adding +0.23 (the minimum SCR value was −0.224 μS).

Among the 33 participants, the grand-average SCR waveforms elicited by the preferred and non-preferred products showed a peak with a latency from 2 to 5 s (Figure 2). In both sessions, the SCR amplitudes elicited by the preferred products were larger compared with those elicited by the non-preferred products. The ANOVA confirmed this visual speculation by indicating a significant main effect of Preference [*F*_(1, 32)_ = 6.52, *p* = 0.0156, η^2^ = 0.17: Figure 3]. However, neither the main effect of Session [*F*_(1, 32)_ = 0.01, *p* = 0.9373] nor the interaction of Preference and Session [*F*_(1, 32)_ = 0.02, *p* = 0.8819] were significant.

**Figure 4:**
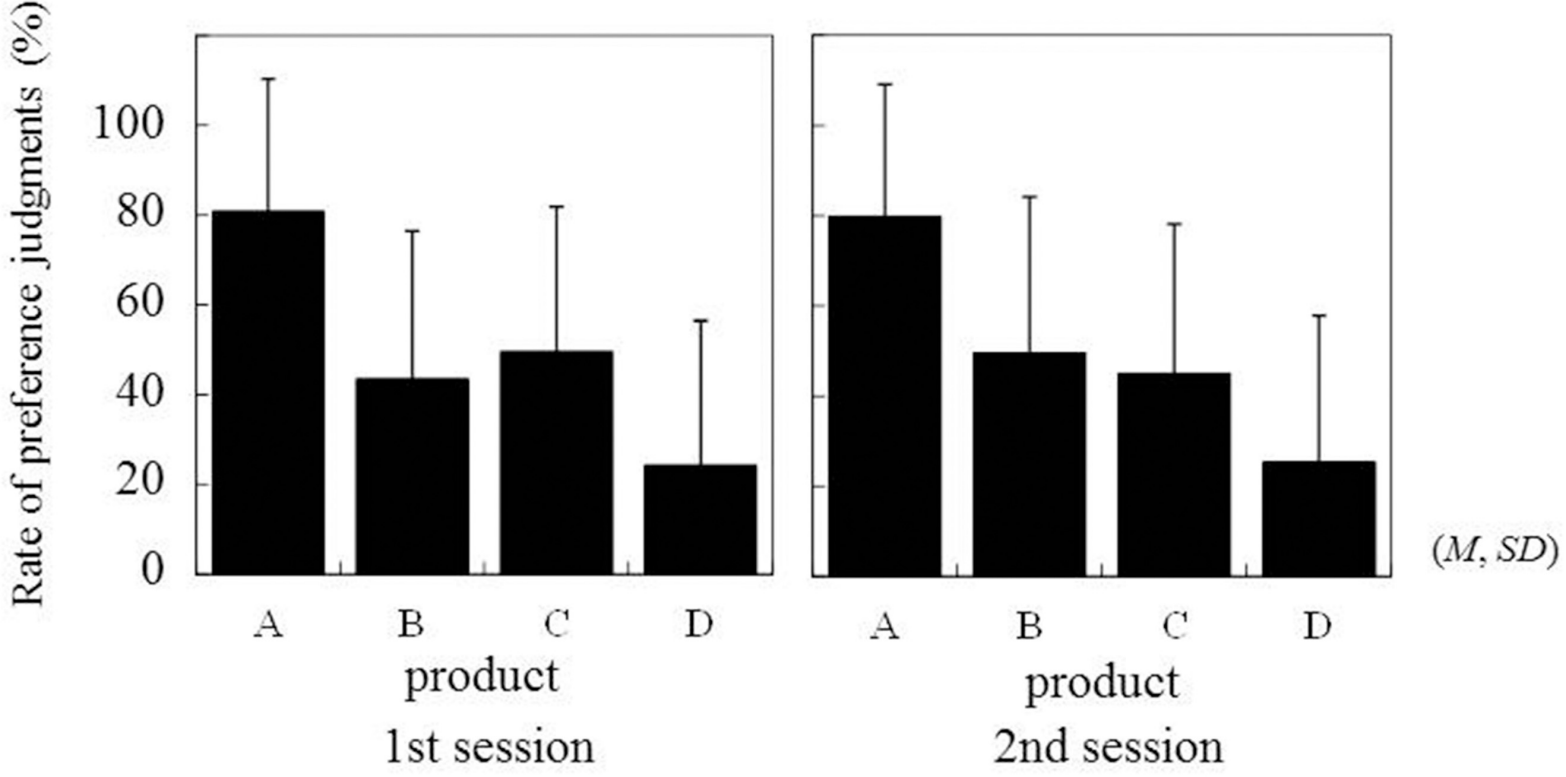
**Rate of preference judgment for each product in two sessions of the paired-comparison task**.

As shown in Figure 4, the order of preference for products was well preserved between the first and second experimental sessions (scale values in Thurstone's paired comparison for product A–D; 0.73, −0.13, 0.01, −0.60 in the first experimental session, and 0.69, 0.00, −0.11, −0.59 in the second experimental session, respectively). The ANOVA for the rates of preference showed only a significant main effect of Product [*F*_(3, 96)_ = 17.25, *p* = 0.0000, η^2^ = 0.35], with no other significant effects (*F* < 0.48, *p* > 0.6984), suggesting that the preferences for the products were reliable. Preferences and SCR values significantly correlated in the first experimental session [*t*_(32)_ = 2.23, *p* = 0. 0332], and a tendency of the correlation was shown in the second experimental session [*t*_(32)_ = 1.14, *p* = 0.0720]. The positive *t*-values in both experimental sessions (2.23 and 1.14) mean that preferences and SCR positively correlated (the more preferred, the larger SCR).

## Discussion

In the present study, we analyzed activity of a physiological index, SCR, during assessing consumer preferences for cosmetic products. While product preference measured using behavioral indexes involves cognitive and affective factors (Bagozzi et al., 1999), physiological indexes, such as SCR, can dissociate those factors of preference (Figner and Murphy, 2011). Specifically, SCR is an index of peripheral α-adrenergic receptor mediated sympathetic activity, and thus is thought to be sensitive to the arousal that accompanies affective or emotional states (Lang et al., 1993; Cuthbert et al., 2000). Consumers might estimate the value of a cosmetic product based not only on logical considerations of function and price but also on vague affective or emotional responses to the item (Guthrie and Kim, 2009). For evaluation of preference for such products, physiological indexes such as SCR should have potential for measuring affective factors.

We measured SCR during a paired-comparison preference judgment task using four cosmetic products as targets. Specifically, we analyzed amplitudes of anticipatory SCR signals produced while participants evaluated one stimulus from a pair as being more or less preferable than the other. As shown in Figure 2, we observed clear SCR peaks 2–6 s after the onset of a stimulus, prior to preference judgment regarding the stimulus. We found that stimuli that were judged as being more preferable elicited larger SCR amplitudes. This result was replicated in our second experimental session, which was conducted 1 year after the first session using the same stimuli and participants, indicating that this finding had robust test-retest reliability. These results suggest that together, the paired-comparison task and SCR measurement can be used to successfully estimate product preference, especially with respect to affective factors.

Our findings appear to be contradictory to those of a previous report, which found that the presentation of disliked brand names elicited a larger SCR compared to liked brand names (Walla et al., 2011). SCR is generally considered to reflect levels of arousal rather than emotional valence (Lang et al., 1993), thus sensitivity of SCR to preferred items and to non-preferred items might be highly task-dependent. While participants in the study by Walla et al. (2011) rated their buying intention on a five-point scale for each brand name in each trial, participants in the present study compared two cosmetic products and judged whether the presented stimulus was more preferable than the absent stimulus in a forced-choice trial. In this type of a situation, decisions about preference may heighten physiological arousal for a preferred item. In our study, participants responded via a button-press to select the preferred product and did not respond to the non-preferred product. Thus, a possible criticism to our finding is that the button-pressing action, rather than the judgment of preference, might elicit a larger SCR (Schacht et al., 2009). However, statistically significant and approaching to significance correlations between preferences of products and SCR amplitudes suggest that SCR measured in the present experimental paradigm can reflect consumers' preferences at least to some degree, but is not merely determined by the button-pressing motor action. This issue should be examined further in more direct ways.

Another possibility is that the physiological arousal reflected by SCR might form an affective state and thus induce feeling of preference for a product. The somatic marker hypothesis (Damasio, 1994) argues that emotional signals associated with bodily and physiological responses are conveyed to the brain, where they contribute to conscious states of positive and negative feelings. SCR has been fundamental in supporting this hypothesis (Bechara et al., 1996, 1997, but also see Dunn et al., 2006). If this is the case, it is possible that a presented image may contain cues regarding prior positive experiences, such as the appearance of product container, the name of the product, and the brand name, can elicit SCR and thus contribute to positive feelings about the product, and finally, preference of the product. This possibility can be further examined to clarify using the processes involved in our experimental paradigm.

There were several limitations to this study. First, the generalizability of the present findings to other kinds of products is not clear; more tests using non-cosmetic products should be conducted. Additionally, we could not control exposure to the cosmetic products used as stimuli in the present study prior to the first experimental session and during the interval between the first and second experimental sessions, as we used real products that were popular in Japan and easily available. Furthermore, as it has been reported that women elicited greater physiological reactivity including SCR than men to emotional materials (Bradley et al., 2001; Chentsova-Dutton and Tsai, 2007), validity of the present findings should be tested also for men. However, despite the above-mentioned limitations, our findings suggest that the combination of the paired comparison and SCR can constitute a simple, robust, and easily accessible index for the evaluation of preferences of commercial products.

### Conflict of interest statement

The authors declare that the research was conducted in the absence of any commercial or financial relationships that could be construed as a potential conflict of interest.
