# Supplementary figures and images for: Analysis of skin conductance response during evaluation of preferences for cosmetic products
Source: Front Psychol. 2015 Feb 9;6:103. doi: 10.3389/fpsyg.2015.00103 (PMC4321331; doi:10.3389/fpsyg.2015.00103)

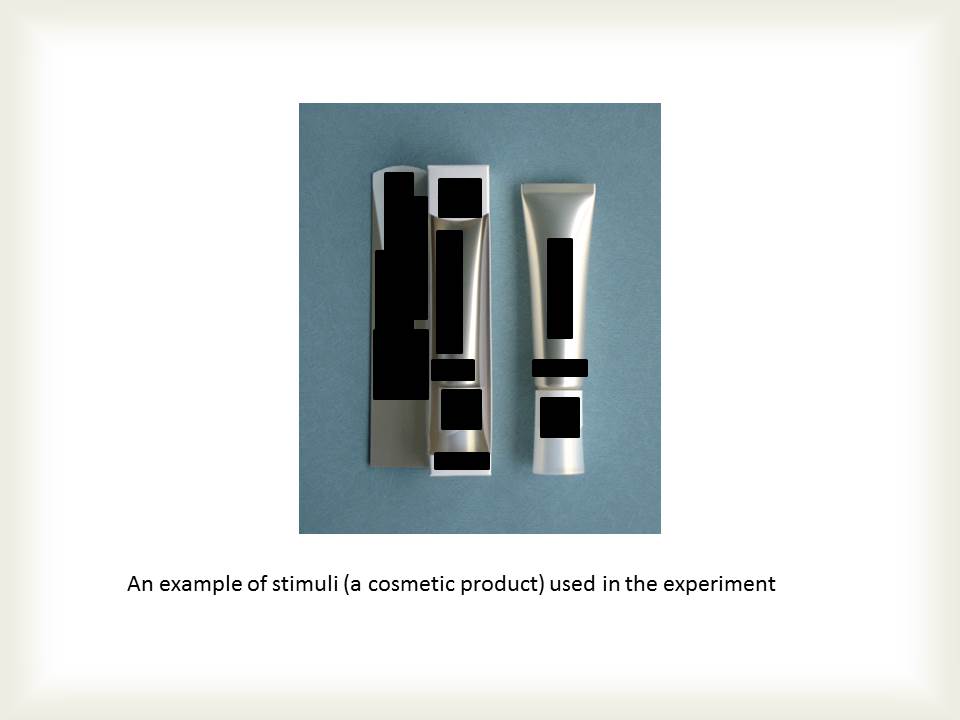

Supplement: Supplementary file 1 [file Image1.JPEG]
